# Lead adsorption by biochar under the elevated competition of cadmium and aluminum

**DOI:** 10.1038/s41598-017-02353-4

**Published:** 2017-05-23

**Authors:** Lu Han, Linbo Qian, Rongqin Liu, Mengfang Chen, Jingchun Yan, Qinhong Hu

**Affiliations:** 1 Key Laboratory of Soil Environment and Pollution Remediation, Institute of Soil Science, Chinese Academy of Sciences, Nanjing, 210008 China; 2 School of Environmental Studies, China University of Geosciences, Wuhan, 430074 China

## Abstract

Competitive adsorption studies are important to accurately estimate the lead adsorption capacity on biochar in soil. The structure of biochars was evaluated by Fourier-Transform Infrared Spectroscopy and X-ray Diffraction, and the competitive of Cadmium (Cd) and Aluminum (Al) with Lead (Pb) adsorption were determined by kinetic experiments and pH effects. Adsorption kinetics indicated that the adsorption amount (mg g^−1^) of Pb by biochar was in the decreasing order of CM400 (90.9) > BB600 (56.5) > CM100 (29.2), the presence of the oxygen-containing functional groups, Si-containing mineral, PO_4_^3−^ and CO_3_^2−^ significantly contributed to Pb adsorption by biochars. With the presence of Cd, Pb adsorption amount was reduced by 42.6%, 23.7% and 19.3% for CM100, CM400 and BB600, respectively. The Si-containing mineral, PO_4_^3−^ and CO_3_^2−^ that were rich in CM400 and BB600 has led to less competition by Cd. In addition, Al showed a strong competition with Pb leading to the adsorption being reduced by 95.8%, 82.3% and 80.6%, respectively for CM100, CM400 and BB600. This was mainly attributed to the additional acidification effect by Al resulting in a counteractive of biochar’s liming effect. Results from this study are important for accurately estimating the heavy metal adsorption by biochar in soil.

## Introduction

Lead (Pb) is a ubiquitous contaminant in soil and aqueous solution environment, which could be removed effectively by adsorption on a range of environmental adsorbents^1–7^. Biochar is a novel sorbent produced by the pyrolysis of a feedstock under oxygen-limited or anaerobic conditions^8–11^. The characteristics of large specific surface area, porous structure, profound surface functional groups and mineral components make it efficient sorbents for multiple contaminants^12–18^. In recent years, many investigations have proved high adsorption capacities of Pb by biochars and the adsorption involves multiple mechanisms, including ion exchange, complexation with oxygen-containing functional groups, precipitation with inorganic components and interactions with π electrons (C=C)^9, 19, 20^. Many studies have been undertaken on the single Pb adsorption by biochars, however, the adsorption ability of biochars is believed to be affected by the coexisting elements, such as other heavy metals, H^+^ or Al^21–26^. Since there are only a very few reports which raised an attention for heavy metal competition on biochar, more intensive studies are needed to evaluate the adsorption capability of biochars under more complicated situations.

Pb is often accompanied by Cd from a range of anthropogenic sources such as batteries, mining and smelting operations^27, 28^. The metal-contaminated soils are usually acidic which inevitably results in Al dissolution^29^. The association and interaction of Pb with Cd and Al can potentially impact the adsorption behavior of Pb by biochars. Recently, some investigations have been performed on the removal of metal ions by biochars in multi-metal systems^30, 31^. Xu *et al*. revealed that the biochar derived from rice husk exhibited a stronger competitive adsorption of metals than that derived from manure due to its limited active sites^32^. Ding *et al*. investigated the competitive removal of Cd and Pb by biochars produced from water hyacinths, indicating that the adsorption process in the mixed solutions of Cd and Pb was more favorable for Pb^31^. Qian *et al*. interpreted that the oxygen-containing functional groups were the major competitive sites between Al and Cd^21^. However, to our knowledge, the competitive behavior of Cd and Al with Pb adsorption by biochar is still unclear, therefore, to study the competitive adsorption mechanism of Al or Cd with Pb by different biochars was quite critical for assessing the amendment of biochar to Pb in soil.

The objective of this study is to investigate the adsorption efficiency of Pb by biochars with Cd or Al. The structure of biochars was evaluated by Fourier-Transform Infrared Spectroscopy (FTIR), X-Ray Diffraction (XRD), Elemental Analyses (EA), BET-surface area (SA), Scanning Electron Microscope (SEM) and zeta potential. The mechanisms responsible for competitive adsorption are elucidated using kinetics models and batch experiments at various pH values.

## Results and Discussion

### The biochar selection

**Figure 1:**
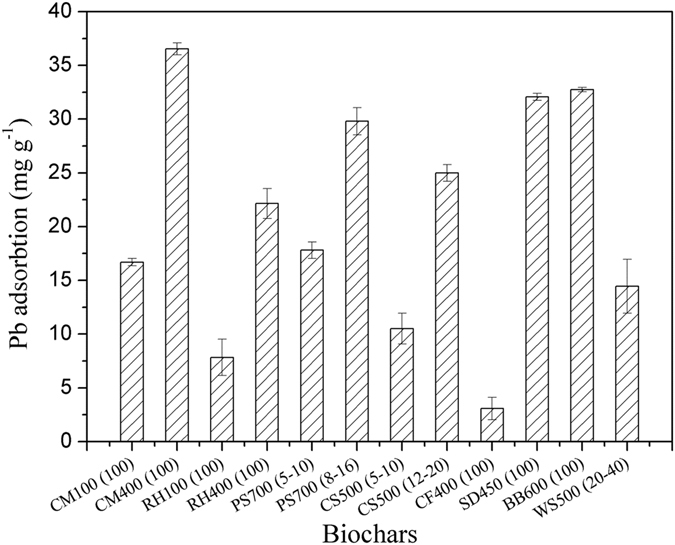
The Pb adsorption amount by different biochars. The capital letters represent the abbreviations of biochar precursors, the number following the letters is the pyrolysis temperature and the number in the bracket is the mesh sieve of biochars being passed through. The initial concentration and pH value were 100 μmol L^−1^ and 3.7, respectively; the biochar loading was 0.25 g L^−1^.

The adsorption amounts of Pb by four laboratory derived biochars and eight commercial biochars are shown in Fig. 1. For the biochar derived from cattle manures and rice husks under the same pyrolysis temperature (100 °C and 400 °C), the biochars derived from cattle manure showed a higher Pb adsorption amount than that derived from rice husks. The adsorption amounts of Pb were 16.7 and 36.2 mg g^−1^ for CM100(100) and CM400(100), respectively. Among the tested samples, CM400(100) was the most effective Pb adsorbent with the adsorption amount much higher than that of RH400(100) (22.1 mg g^−1^). This is mainly attributed to the higher phosphorite containing in cattle manure^30^. In addition, the Pb adsorption amount by biochar produced at 400 °C (CM400(100) and RH400(100)) was significantly higher than those pyrolyzed under 100 °C (CM100(100) and RH100(100)) (Fig. 1). Previous investigation on the Al adsorption by biochar derived from cattle manures and rice straws revealed that the biochars pyrolyzed at 400 °C was the most effective Al adsorbent, as the oxygen-containing organic components and the scattering of silicate particles were responsible for Al adsorption^13^. The Pb adsorption amounts by commercial biochars ranged from 3.1 to 32.8 mg g^−1^, which was sample dependent. The highest Pb adsorption amount was the biochar derived from banboo (BB600(100)). Thus BB600(100) was selected as the representative of commercial biochars. Eventually, three typical types of biochars, CM100(100), CM400(100) and BB600(100) (here after referred as CM100, CM400 and BB600) were selected for further evaluations.

### The structure characterization of the selected biochars

**Figure 2:**
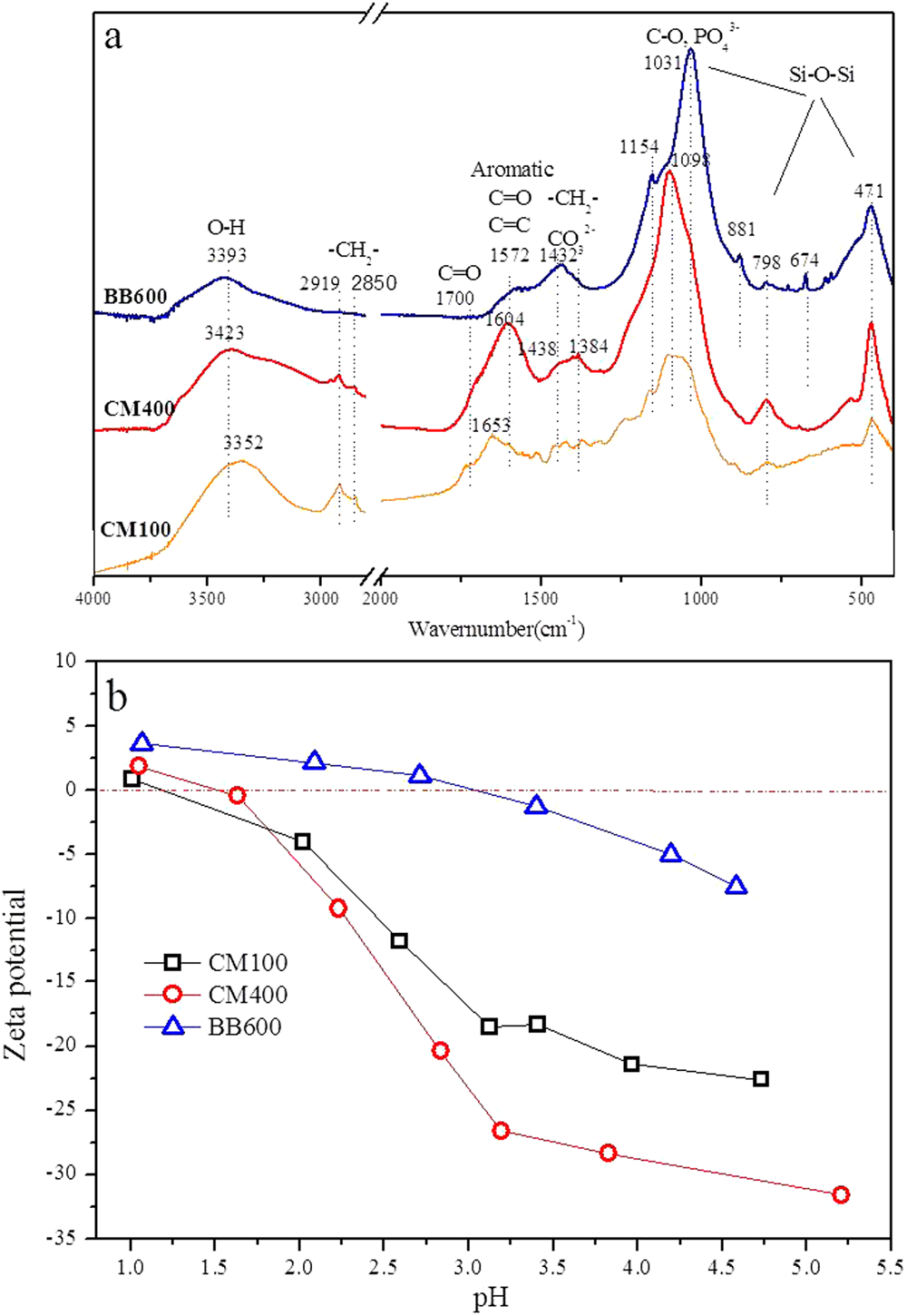
FTIR spectra (**a**) and ζ-Potential (**b**) of the cattle manure biochar (CM100 and CM400) and bamboo biochar (BB600). The numbers in the sample names represent the pyrolysis temperatures.

The FTIR spectra of CM100, CM400 and BB600 are illustrated in Fig. 2a. It can be seen that the spectrum of CM100 was characterized by the highest band intensities for the organic functional groups with a band of -OH stretching (3352 cm^−1^), aliphatic CH_2_ (2919, 2850, 1438, 1432 and 1384 cm^−1^), C=O stretching of carboxyl (1700 cm^−1^), aromatic C=O or C=C ring stretching (1653 cm^−1^), C-O stretching (1031 and 1098 cm^−1^) and Si-O-Si stretching (1154–1031, 798, and 471 cm^−1^)^10, 13, 33^. All these bands varied with increasing pyrolysis temperatures. When heating to 400 °C, the band intensities of aliphatic −OH, −CH_2_− and C=O of carboxyl were slightly decreased, which was attributed to the decomposition of chain hydrocarbon and rearrangement of molecules at high temperatures. The band intensity of aromatic C=O or C=C of CM400 was dramatically increased, suggestive of a decrease of nonpolar aliphatic and an increase of aromatization. In addition, the band intensity of Si-O-Si was shown to increase significantly, inducing a high silicon content from its original feedstock. The high content of silicon in cattle manures and its derived biochar was previously reported^13^. It is also interesting to note that the Si-O-Si and oxygen-containing groups were observed in the commercial biochar BB600. Additionally, the peaks of PO_4_^3−^ (1098 cm^−1^ and 1031 cm^−1^) and CO_3_^2−^ (1438 cm^−1^ and 1432 cm^−1^) were observed in CM400 and BB600^30^.

Figure 2b illustrates the pH-dependent ζ-potential curves of the CM100, CM400, and BB600 samples. The pH at the isoelectric point (pH_IEP_) for CM100 and CM400 were less than 2.0. In contrast, the pH_IEP_ was increased to approximately 3.2 for BB600 pyrolyzed at high temperatures. The biochar particles will carry negative surface charges in the case of the solution pH > pH_IEP_. The surface charge of the biochars are controlled by both the organic components (−COOH− and −OH) and mineral fraction potentially^13^.

**Figure 3:**
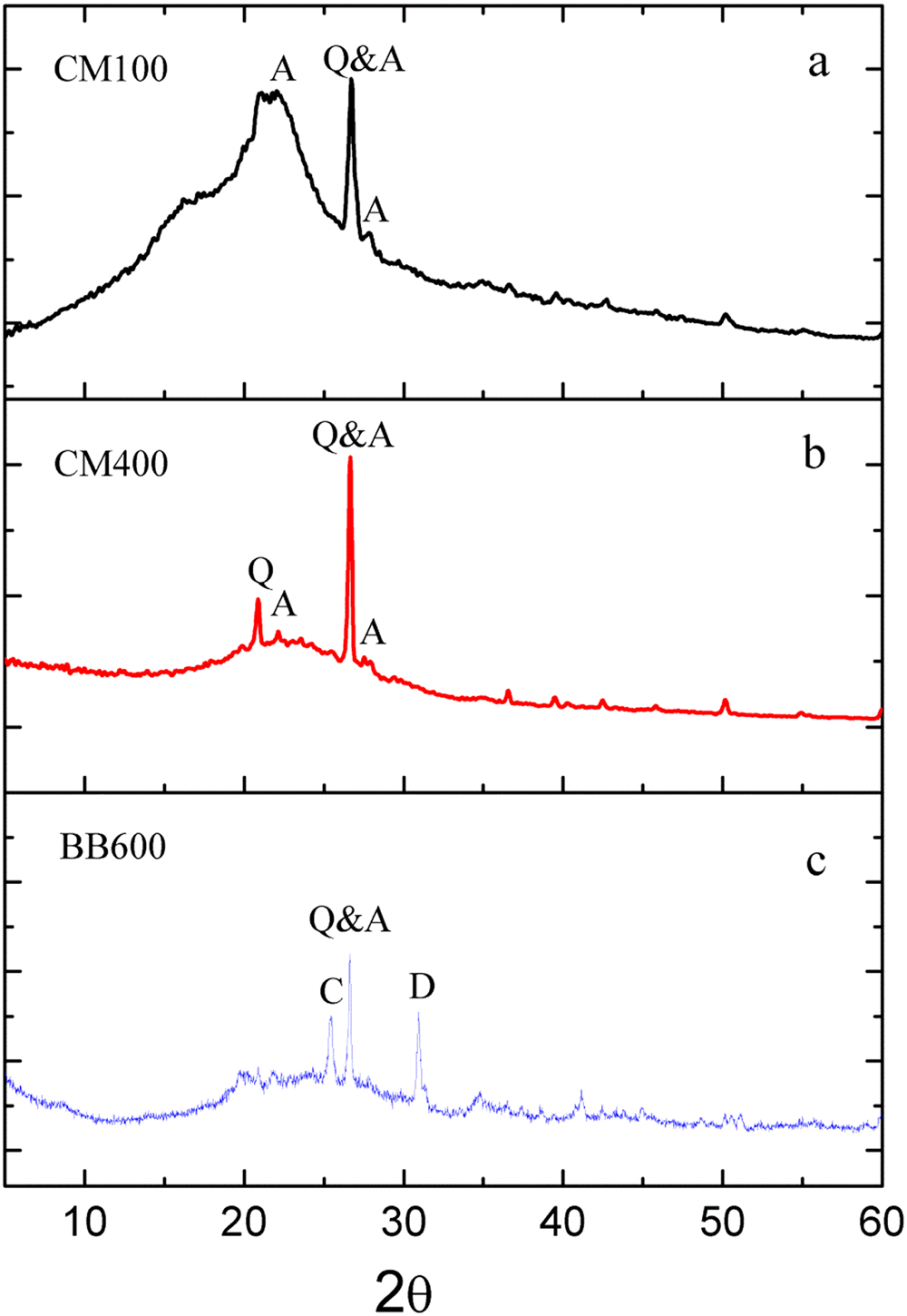
X-ray diffraction patterns of the cattle manure biochar (CM100 and CM400), Bamboo biochar (BB600). The numbers in the sample names represent the pyrolysis temperatures. The following minerals are labeled with respect to their peaks: A, albite; C, calcium sulfate; D, dolomite and Q, quartz.

The XRD spectra of biochar samples are shown in Fig. 3. Similar to the changes of organic functional groups of biochar samples, the crystal minerals of biochars were varied as the pyrolysis temperature was increased. For CM100, more amorphous organic components were found and only the presence of albite was confirmed^13^. However, when the pyrolysis temperature was increased to 400 °C, the amorphous organic components were gradually diminished and the crystal mineral were formed. New diffraction peaks for calcium sulfate and dolomite were appeared for BB600. The presence of albite and quartz indicated that the original feedstocks were rich in Si which can be manifested by Si-O-Si stretching band from FTIR spectra (Fig. 2a). The dolomite was probably the major alkaline minerals present in BB600.

The pH values and mineral element analysis of CM100, CM400 and BB600 are detailed in Table 1. All biochar samples were at alkaline pH values, with the potential to neutralize the acidity when used as amendments. The difference in pH may be arisen from different pyrolysis temperatures and feedstock types^34^. The elemental analysis showed that biochar derived from the cattle manure had higher contents of K and Ca than bamboo-based biochar. However, more mineral components especially for Mg can be found in BB600, being approximately four times higher than that of CM400 (1.67% vs. 0.41%). The mineral elemental analysis corresponded favorably with the crystal mineral analysis by XRD. The content of P in biochars was significantly increased with the increase of the pyrolysis temperature. The presence of these mineral components in biochars pyrolyzed under high temperature was important in processes involving complexation and precipitation with the metal components. Overall, the variation of biochar surface characteristics, regulated by the pyrolysis condition and properties of the original feedstock, would have significant impacts on their adsorption capacity and removal mechanism.

**Table 1:** The pH values and the elemental analysis of the cattle manure biochar (CM100 and CM400) and bamboo biochar (BB600).

Elements	CM100	CM400	BB600
pH (1:20)	6.95	8.59	7.92
K (%)	0.54 ± 0.03	1.52 ± 0.01	0.65 ± 0.04
Na (%)	0.05 ± 0.00	0.14 ± 0.00	0.09 ± 0.00
Ca (%)	0.47 ± 0.01	1.28 ± 0.02	1.35 ± 0.09
Mg (%)	0.15 ± 0.00	0.41 ± 0.00	1.67 ± 0.10
P (%)	0.34 ± 0.00	0.59 ± 0.01	0.74 ± 0.04

The numbers in the sample names represent the pyrolysis temperatures.

**Figure 4:**
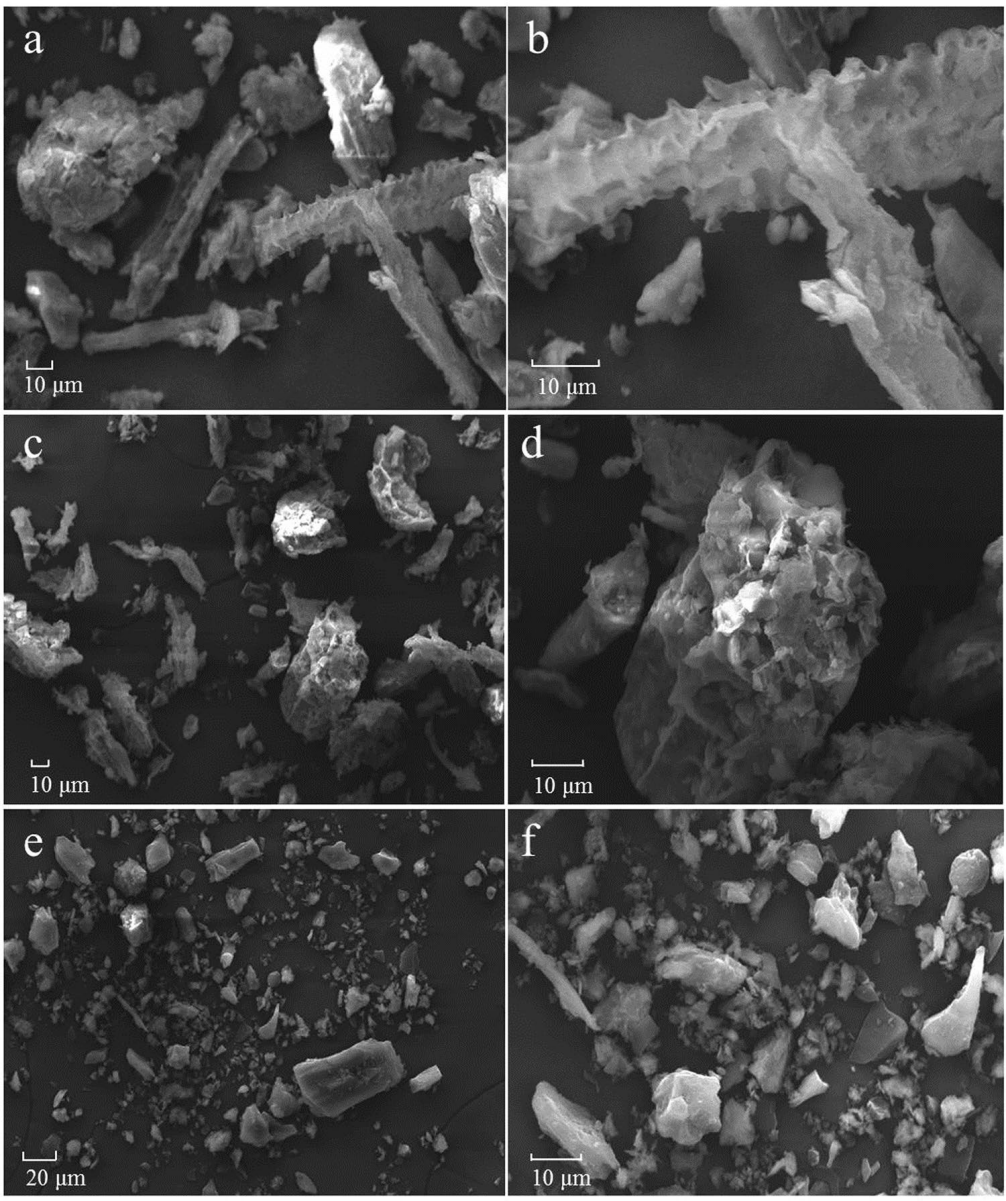
Scanning electron micrograph spectra of the biochar produced from cattle manure [CM100 (**a**,**b**) and CM400 (**c**,**d**)], Bamboo biochar [BB600 (**e**,**f**)]. The numbers in the sample names represent the pyrolysis temperatures.

Figure 4 shows the SEM micrographs of CM100, CM400 and BB600. The surface of the CM100 was clean (Fig. 4a,b), indicating an incomplete pyrolysis of the biomass, whereas, more mineral fractions were present on the surface of CM400 (Fig. 4c,d) due to the decomposing of organic components decomposing as the pyrolysis temperature being increased. The structural morphology of BB600 (Fig. 4e,f) was relatively compact and some impurities were present in the commercial biochar.

The data of surface area, pore volume and adsorption average pore diameter is presented in Table 2. BB600 showed the greatest surface area (447.46 m² g^−1^) and total pore volume (524.17 mm³ g^−1^), followed by CM400 and CM100. They mainly contained mesopores (2–50 nm) according to the average pore diameter data (4.69~9.12 nm).

**Table 2:** Surface area and porous structure of biochars.

Biochar	BET-SA (m² g^−1^)	t-Plot MA (m² g^−1^)	TPV (mm³ g^−1^)	t-Plot MV (mm³ g^−1^)	APD (nm)
CM100	7.78	2.43	12.37	0.24	6.36
CM400	11.64	1.88	26.54	0.17	9.12
BB600	447.46	203.54	524.17	65.27	4.69

BET-SA: BET-surface area; t-Plot MA: t-Plot micropore area; TPV: total pore volume; t-Plot MPV: t-Plot micropore volume; APD: adsorption average pore diameter (4 V/A by BET).

### Effect of the coexisting Cd and Al on Pb adsorption kinetics

**Figure 5:**
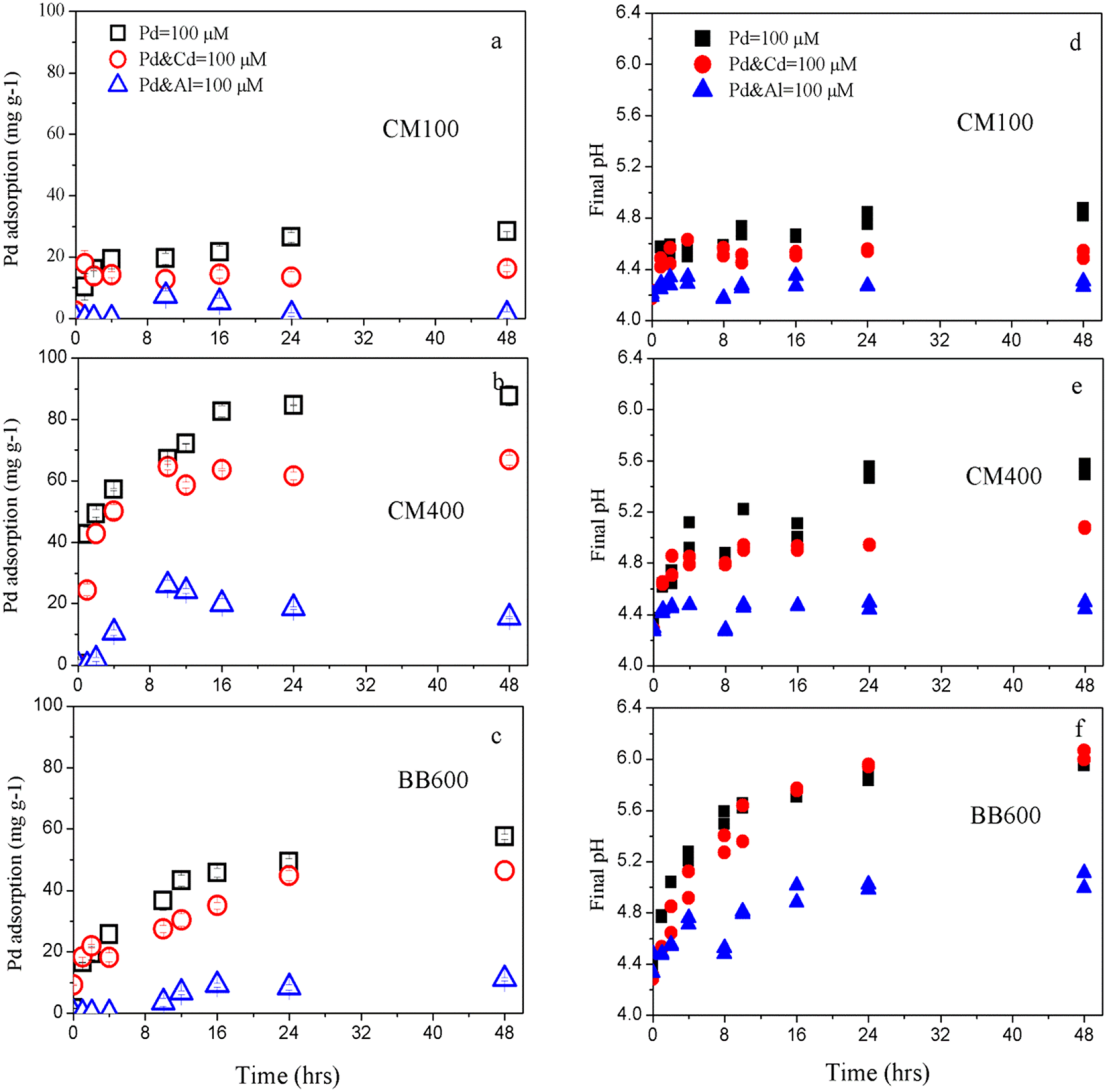
Effect of coexisting Cd and Al on Pb adsorption kinetics onto the cattle manure biochar [CM100 (**a**) and CM400 (**b**)] and bamboo biochar [BB600 (**c**)], the final pH of the cattle manure biochar [CM100 (**d**) and CM400 (**e**)] and bamboo biochar [BB600 (**f**)] after adsorption. The numbers in the sample names represent the pyrolysis temperatures.

The kinetics of Pb adsorption, with/without Cd and Al are illustrated in Fig. 5, and the kinetic parameters derived from model simulations are given in Table 3. The adsorption kinetics of Pb were well fitted better by the pseudo second-order model (R^2^ = 88.6–98.1%) than the pseudo first-order model (R^2^ = 64.9–97.5%) for the single-metal system. The adsorption of Pb by biochars can be divided into fast and slow processes. In the first 4 hrs, Pb was quickly adsorbed by CM100, CM400 and BB600, which were accounted for 67.8%, 65.2% and 44.7% of their total Pb adsorption amount, respectively. Then, the amount of Pb adsorption was gradually increased during the next 44 hrs. It is also illustrated in Table 3 that the adsorption of Pb by both CM400 and BB600 was slower than that by CM100. Since biochar contains many heterogeneous components, the fast- and slow-adsorption processes may be strongly affected by the activation of these components. The organic components were contained in CM100 mainly and they may be likely to interact with Pb quickly. As the pyrolysis temperature was increased, more mineral fractions in CM400 and BB600 were exposed to the Pb adsorption, leading to slow the adsorption kinetics. The pH increases during the Pb adsorption implied that the activation of these components was probably regulated by pH values (Fig. 5d–f).

**Table 3:** Parameters of pseudo first- and second-order kinetic models for Pb adsorption by the cattle manure biochar (CM100 and CM400) and bamboo biochar (BB600).

Biochar	Pseudo first-order model			Pseudo second-order model		
	*Q* (mg g^−1^)	*k*_*1*_ × 10^2^ (h^−1^)	*R* ^2^	*Q* (mg g^−1^)	*k*_2_ × 10^2^ (g mg^−1^ h^−1^)	*R* ^2^
CM100	28.2	3.39	0.98	29.2	2.88	0.97
CM400	79.1	2.28	0.65	90.9	0.38	0.87
BB600	50.5	3.97	0.98	56.5	0.73	0.98

The numbers in the sample names represent the pyrolysis temperatures.

Among the three biochars, CM400 is showed to be the most effective for the adsorption amounts of Pb with 90.9, 56.5 and 29.3 mg g^−1^, respectively for CM400, BB600 and CM100. The adsorption amount of Pb by CM400 was slightly lower than that by the sesame straw biochar pyrolysis under 700 °C (102 mg g^−1^) recently reported by Park *et al*.^35^, but much higher than that by the orchard pruning-derived biochar pyrolysis under 500 °C (22.42 mg g^−1^) reported by Caporale *et al*.^36^. This phenomenon was consistent with the findings of Kolodynska *et al*.^37^, who proposed that the biochar pyrolysis at 400 °C appears to be effective for heavy metal adsorption, particularly for the highest adsorption capacity for Pb (20.72 mg g^−1^) by pig manure based biochar. The oxygen-containing functional groups (-OH and -COO-) and mineral fractions including CO_3_^2−^, SiO_3_^2−^ and PO_4_^3−^ were present simultaneously in CM400 (Fig. 2), consistent with the documented literatures^7, 13, 38^. It is attributable that the two dual roles including both oxygen-containing organic components and mineral components contribute to its effective adsorption capability^13^. Higher pH values may be another significant factor to improve the potential adsorption capability of Pb^39^. The kinetics data indicated that the adsorption of Pb by biochars was rate-limited chemisorption which may be related to the pH-dependent release of active sites.

As illustrated in Fig. 5a–c, the adsorption of Pb by biochars was inhibited with the presence of Cd. In the first 4 hrs, the effect of Cd on Pb adsorption was limited, however, the adsorption rate and the maximum adsorption amount of Pb were notably decreased in the next 44 hrs. In the presence of Cd, the adsorption amount of Pb by CM100, CM400 and BB600 were decreased by 42.6%, 23.7% and 19.3%, respectively. It may be interpreted as the competitive adsorption taking place for the same binding sites such as oxygen-containing functional groups, mineral components, PO_4_^3−^ and CO_3_^2−^ ^21, 30^. The competitive adsorption process of Cd and Pb was different from its corresponding behavior in Pb adsorption by biochar alone, since the pseudo second model was no longer suitable for describing its adsorption process. This phenomenon indicated that the mechanism of metal ions competitive adsorption by biochars was complex and probably a combination of many sorption processes.

The presence of Al appeared to exhibit a stronger competition than Cd. As shown in Fig. 5a–c, the Pb adsorption by biochar were completely inhibited in the presence of Al, slow adsorption rate and less adsorption amount were observed. The maximum adsorption amount of Pb by CM100, CM400 and BB600 was significantly decreased by 95.8%, 82.3%, 80.6%, respectively. Apart from the competition for the same sites, the acidification effect of Al may be another significant factor to inhibit the adsorption of Pb, as the finial pH of CM100, CM400 and BB600 were greatly decreased (4.3, 4.5 and 5.0, respectively) compared with the situation by Pb adsorption alone (Fig. 5d–f). Previous study reviewed that the Al competition largely modifies the Pb^2+^ in solution and reduced the amount of Pb^2+^ bound^29^. It was suggested that the competition effect was increased with a decreasing pH value. For example, compared to Cd, the final pH of CM100, CM400 and BB600 was greatly decreased to 4.52, 5.08 and 6.03 in case of Cd to 4.29, 4.47 and 5.05 in the presence of Al. Therefore, the present of Al showed a stronger inhibition of Pb adsorption than that of Cd.

The competition capability of Cd and Al with Pb adsorption on biochars were decreased by CM100 > CM400 > BB600. It is identified that the contents of the organic components were decreased in CM100, CM400 and BB600 gradually (Fig. 2a), as the mineral fractions were increased. Therefore, the increasing mineral fractions were likely to lead the specific adsorption with Pb in resisting the competition of Cd and Al more effectively.

### Effect of coexisting Cd and Al on Pb adsorption under different pH conditions

**Figure 6:**
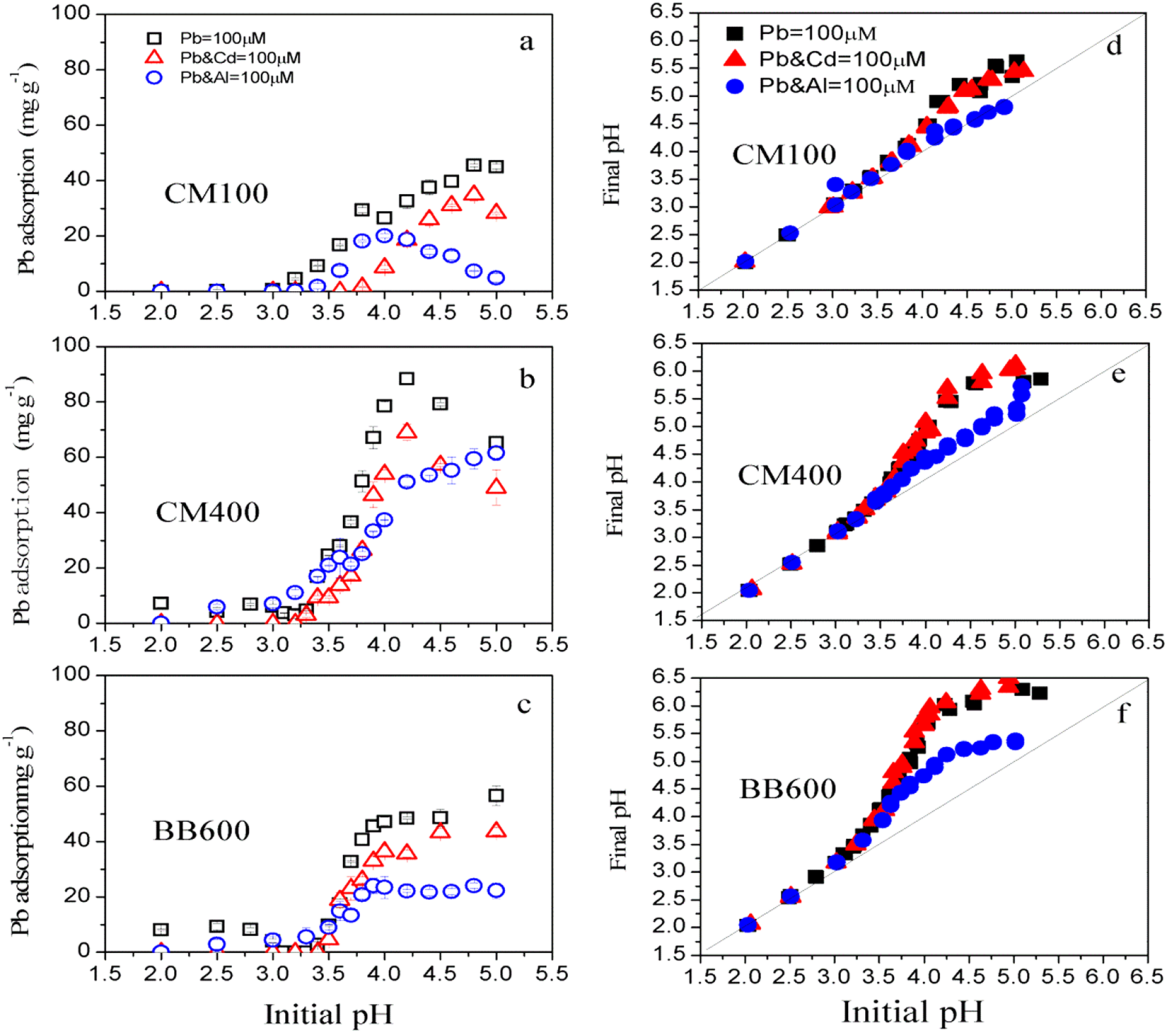
Effect of coexisting Cd and Al on Pb adsorption under different pH conditions onto the cattle manure biochar [CM100 (**a**) and CM400 (**b**)] and bamboo biochar [BB600 (**c**)], the final pH of the cattle manure biochar [CM100 (**d**) and CM400 (**e**)] and bamboo biochar [BB600 (**f**)] after adsorption. The numbers in the sample names represent the pyrolysis temperatures.

The pH-dependent adsorption of Pb in aqueous solution by biochars are shown in Fig. 6. When the initial pH was below 3.0, little Pb adsorption by CM100 was observed. Compared to CM100, the Pb adsorption by CM400 and BB600 were significantly increased. At initial pH3.0, the Pb adsorption amount was 6.1 mg g^−1^ and 4.1 mg g^−1^ for CM400 and BB600, respectively. When the initial pH was increased to 3.6, Pb adsorption by biochar was enhanced with the adsorption amount being 16.7 mg g^−1^ for CM100 and 27.9 mg g^−1^ and 17.6 mg g^−1^ for CM400 and BB600, respectively. When the initial pH value was above 3.6, a positive correlation between the rise of initial pH and the amount of Pb adsorption was observed. The highest Pb adsorption by CM100 (45.0 mg g^−1^), CM400 (88.3 mg g^−1^) and BB600 (56.5 mg g^−1^) were observed at initial pH5.0, pH4.2 and pH5.0, respectively. pH is an important parameter during the adsorption process, and many investigations have approved that the Pb adsorption was pH-dependent^37, 40^. The pH value can not only affect the speciation of metal ions, but also the ability to form surface electrical charges on the biochars^13, 41^. At lower pH, the competition of Pb with H^+^ occurred which makes the lead ions inaccessible to the surface functional groups (mainly oxygen-containing groups)^42^. When the initial pH was increased, the deprotonation of functional groups and the dissolution of some specific mineral components can potentially provide more active sites for Pb, resulting in an increase of adsorption effectiveness^43^. As demonstrated by many studies, the activation of these components was dependent on the solution pH. For example, carboxyl groups become deprotonated when the solution pH was above 3.7, these groups would be dissociated to provide more active sites for heavy metal adsorption^4, 5, 44^. In addition, SiO_2_ component is negatively charged until the pH is increased to 4.5^13, 45^, but it can also contribute to the adsorption of heavy metals when the pH is above 4.5. Therefore, the increase of the initial pH could make these adsorption sites being activated and completely occupied by heavy metals. The rise of pH would also be favorable for the Pb precipitation by PO_4_^3−^ and CO_3_^2−^ ^46^.

A competition behavior with the presence of Cd for the three selected biochars is illustrated in Fig. 6a–c. The Pb adsorption by CM100, CM400 and BB600 were completely inhibited when pH value was lower than 3.0. The adsorption of Pb was then increased when the initial pH was above 3.0, but the amount was significantly lower than that without Cd. The highest Pb adsorption by CM100, CM400 and BB600 was observed at initial pH4.8, pH4.2 and pH5.0, respectively under the present experimental conditions. It can be interpreted that the Cd was involved in the competition of Pb adsorption sites such as oxygen-containing groups and minerals (3.0–4.4). Additionally, anions such as PO_4_^3−^ and CO_3_^2−^ which were shown to precipitate with heavy metals will also be dissolved^38, 47^. As CM100 contains rich oxygen-containing functional groups but little mineral components, strong affinity for Pb was identified during the dissociation of oxygen-containing groups at pH 3.8–4.5. CM400 appeared to have a wider competitive pH ranges from 3.0 to 5.0, in which the dissociation of oxygen-containing groups and dissolution of minerals took place at the pH 3.0–4.5 and 4.0–5.0 respectively. Comparatively, BB600 with more mineral components but little oxygen-containing functional groups, which lead to the Pb adsorption to the dissolution mineral at the pH 3.5–5.0. The highest Pb adsorption by CM100 and CM400 was reached at pH4.8 and pH4.2, respectively, and then the adsorption amount was decreased. This was mainly because that the actual concentration of Pb in solution was decreased with pH value increasing, and the adsorption amount of Pb was calculated on the basis of the determined initial and equilibrium concentration of Pb in solution, leading to the adsorption amount increasing at first then decreasing (Fig. 6d–f). It was mainly caused by the Pb precipitation at a high pH value.

When Al was present as a competing ion with Pb, the competitive effect was quite different from the Cd coexistence (Fig. 6a–c). When the pH was below 3.5, little Pb adsorption by CM100 was observed. When the pH was increased from 3.5 to 4.0, the Pb adsorption by CM100 was progressively increased, the highest Pb adsorption amount was reached at initial pH4.0. Then, as the initial pH was further increased, the Pb adsorption by CM100 was sharply decreased with nearly 100% reduction being observed when initial pH was 5.0. The presence of Pb^2+^ and AlOH^2+^ in aqueous solution for pH 4.0–5.0, indicated the higher adsorption of AlOH^2+^ than Pb^2+^ by CM100. Previous study on aluminum competition for lead and cadmium binding to humic acids showed that the effect of aluminum on the lead binding is important with the amount of lead binding being reduced by a factor of 2 to 3^29^.

Interestingly, the Pb adsorption by CM400 and BB600 was progressively increased as the initial pH was increased. This is mainly due to the more adsorption sites being available within CM400 and BB600, including oxygen functional groups, Si-containing components, PO_4_^3−^ and CO_3_^2−^. Additionally, the lowest Pb adsorption amount was observed with the coexistence of Al, and this was mainly attributed to the pH buffering capability being completely inhibited. The hydrolysis of Al that led to strong bond with OH^−^ and the release of more H^+^ may be the main reason for stronger competition of Al than Cd during the process of adsorption kinetics.

The effects of Cd and Al coexistence on Pb adsorption illustrated that the competition intensity was in the order of CM100 > CM400 > BB600. The strongest competition for the adsorption of metals by CM100 was due to the fact that all metals competed only for ionized oxygen-containing groups, but the less competition for the adsorption of metals by CM400 and BB600 were identified not only from the ionized oxygen-containing groups but also from the adsorption and precipitation of metals with Si-containing mineral, PO_4_^3−^ and CO_3_^2−^ that were rich in CM400 and BB600. This study also indicates that the competition of metals on ionized oxygen-containing groups was stronger than that on Si-containing mineral, PO_4_^3−^ and CO_3_^2−^. For the effect of different metals on Pb adsorption, Al showed a strong competition with Pb. This was mainly attributed to the additional acidification effect by Al leading to a counteractive of biochar’s liming effect.

## Conclusions

The objective of this research was to evaluate the competition of Cd and Al with Pb adsorption by biochars. Adsorption kinetics indicated that the adsorption capacity (mg g^−1^) of Pb by biochars were in the order CM400 (90.9) > BB600 (56.5) > CM100 (29.2), and the presence of the oxygen-containing functional groups, Si-containing components, PO_4_^3−^ and CO_3_^2−^ significantly contributed to the adsorption of Pb by biochars. A competitive effect was observed in the presence of Cd with the adsorption of Pb being reduced by 42.6%, 23.7% and 19.3% for CM100, CM400 and BB600, respectively. More significantly, Al was shown to have a strong competition with Pb leading to the adsorption being reduced by 95.8%, 82.3% and 80.6%, respectively for CM100, CM400 and BB600. The acidification effect by Al leads to a counteractive of biochar’ liming effect and the competition by Cd and Al would be curial factors in reducing the available adsorption sites.

## Materials and Methods

### Preparation of biochar

Twelve biochar samples were collected, eight of which were commercially produced with the remaining four samples derived from cattle manure and rice husk in the laboratory. Cattle manures were collected from a local farm located in Anqing, Anhui Province, China and rice husks were collected from the Changshu Agroecological Experiment Station of Institute of Soil Science, Chinese Academy of Sciences. The preparation was made in accordance with the modified procedure^33^. The biomasses were air-dried for 2 days and grounded through 100 mesh sieve. In order to prevent the presence of oxygen during calcination, the feedstocks were compacted in a ceramic pot (about 100 g) and pyrolyzed under the oxygen-limited conditions for 6 h under the temperatures of 100 °C and 400 °C. The heating rate was controlled at 5 °C min^−1^ to reach the target temperature. Finally, the obtained biochars were grounded and passed through 100 mesh sieve before use. The commercial biochars were produced from the peach shell (PS700(5–10) and PS700(8–16)), the coconut shell (CS500(5–10) and CS700(12–20)), the Chinese fir (CF400(100)), the saw dust (SD450(100)), the bamboo (BB600(100)) and the walnut shell (WS500(20–40)). In the sample naming convention, the first two capital letters represents the abbreviations of sample sources, the number following the sample source indicates the pyrolysis temperature and the number in the bracket represents the mesh sieve size or a range of mesh sieve sizes of biochar being passed through. For example, WS500(20–40) refers to the biochar produced from the walnut shell and pyrolyzed under the temperature of 500 °C that went through the mesh sieve sizes from 20 to 40.

### Characterization of biochars

The biochars selected from twelve biochars were characterized by Fourier-Transform Infrared Spectroscopy (FTIR), X-Ray Diffraction (XRD), Elemental Analyses (EA), BET-surface area (SA), Scanning Electron Microscope (SEM) and zeta potential. FTIR was performed by mixing biochar samples with KBr at a ratio of 1:100 (w/w). The spectra were collected in the range from 400 to 4000 cm^−1^ wavenumber at 1 cm^−1^ resolution with 64 scans by FTIR spectrophotometer (Thermo Scientific 7600, USA). XRD (RIGAKU D/MAX 2550/PC, Japan) was operated at 40 kV and 40 mA. The data were collected at the range (2θ) of 2 to 60° using Cu Kα radiation with a scan step of 0.02° and at a rate of 2° min^−1^. The phase peaks were identified by comparing the observed XRD patterns to standards compiled by the Joint Committee on Powder Diffraction and Standards (PDF22004). ASAP 2020 BET-surface area analyzer was used to measure the surface and porous structures of biochars (Micromeritics, Norcross, GA, USA). The morphology of biochars was determined using SEM (ZEISS EVO18, Germany). The surface charge properties of the biochars were evaluated by ζ-potential measurements at different equilibrium pH values with a Nano-ZS90 Zetasizer (Malvern Instruments).

To analyze the mineral element contents (K, Ca, Na, Mg and P) in biochars, the biochar samples were digested in the mixture of HNO_3_, HF and HClO_4_ for 24 hrs at 150 °C. Then K, Ca, Na and Mg were analyzed by Inductively Coupled Plasma-Atomic Emission Spectroscopy (ICP-AES, optima 8000), and the total P was determined by the ascorbic acid-NH_4_-molybdate blue colorimetry at 680 nm. The pH of biochars was measured by Orion Star A214 pH/TSE meter in deionized water at the ratio of 1:20 w/v after shaking for 24 hrs at 150 rpm^34^.

### Batch adsorption experiments

The biochar selection experiment, the kinetic removal experiments and pH dependent removal experiments were performed in this study. The biochar selection experiment was to screen the biochar showing a higher adsorption capability on the basis of the uniform initial concentration. It was conducted in 10 mL glass tube by mixing 2 mg of biochars with 8 mL of 0.5 mmol L^−1^ CaCl_2_, which was used to simulate the soil electrolyte solution (containing 100 μmol L^−1^ Pb (PbCl_2_)^33^ with the initial pH value of 3.7. The pH values were adjusted with 0.1 M NaOH and/or HCl solutions. All the mixture was shaken at 150 rpm under 25 °C. Then, the solid residue and liquid phase were separated by filtering through a 0.45-μm millipore filter. The filtrate was immediately acidified by 0.2% (v/v) HCl for the determination of Pb by Atomic Absorption Spectroscopy (AAS, Varian spectra AA220).

The adsorption kinetics of Pb at an initial pH value of 4.0 (to avoid metal ion precipitation) was investigated. The experiments were conducted with and without the presence of Cd (CdCl_2_·2.5H_2_O) or Al (AlCl_3_·6H_2_O). The kinetic experiments were conducted in both monometal and bimetal system. 8 mg of biochars were added to 40 mL of 0.5 mmol L^−1^ CaCl_2_ containing the adsorbate in a 50-mL polypropylene tube. The adsorbate was 100 μmol L^−1^ of Pb only in the monometal system. Competitive adsorption of Pb and Cd, or Pb and Al were contained in the bimetal system, in which the concentration of individual metal ion was 100 μmol L^−1^. The samples were analyzed at time intervals of 0, 1, 2, 4, 8, 10, 12, 16, 24, 48 hrs. After that, the concentration of Pb was determined by AAS.

The pH-dependent of Pb adsorption experiments were undertaken by adjusting the initial pH values between 2.0 and 5.0 shaking for 24 hrs; the test was conducted in the same procedure as the kinetic adsorption experiments except for the initial solution pH and contacting time. The final pH of all batch adsorption experiments was measured. All three types of adsorption experiments were undertaken in duplicate and with appropriate blanks.
